# The development of the Comprehensive Analysis of Policy on Physical Activity (CAPPA) framework

**DOI:** 10.1186/s12966-019-0822-5

**Published:** 2019-08-02

**Authors:** Bojana Klepac Pogrmilovic, Grant O’Sullivan, Karen Milton, Stuart J. H. Biddle, Adrian Bauman, William Bellew, Nick Cavill, Sonja Kahlmeier, Michael P. Kelly, Nanette Mutrie, Michael Pratt, Harry Rutter, Andrea Ramirez Varela, Catherine Woods, Zeljko Pedisic

**Affiliations:** 1 Institute for Health and Sport, Victoria University, Ballarat Road, Footscray, Melbourne, VIC 3001 Australia; 2 Norwich Medical School, University of East Anglia, Norwich Research Park, Norwich, Norfolk, NR4 7TJ UK; 3 Institute for Resilient Regions, University of Southern Queensland, 37 Sinnathamby, Boulevard, Springfield Central, QLD 4300 Australia; 4 Sydney School of Public Health, University of Sydney, Camperdown, Sydney, NSW Australia; 5 Cavill Associates, Stockport, UK; 6 Department of Health, Swiss Distance University of Applied Science FFHS, Regensdorf/Zurich, Switzerland; 7 Department of Public Health and Primary Care, Institute of Public Health, University of Cambridge, Cambridge, CB2 0SR UK; 8 Moray House School of Education, Physical Activity for Health Research Centre, University of Edinburgh, Edinburgh, Scotland, UK; 9 University of California San Diego Institute for Public Health, 9500 Gilman Drive, San Diego, USA; 10 Department of Social and Policy Sciences, University of Bath, Claverton Down, Bath, BA2 7AY UK; 11 Post-Graduate Program in Epidemiology, Federal University of Pelotas, Pelotas, Brazil; 12 Faculty of Medicine, University de los Andes, Bogota, Colombia; 13 Physical Activity for Health Research Cluster, Health Research Institute, Department of Physical Education and Sport Sciences, Faculty of Education and Health Sciences, University of Limerick, Luimneach, Ireland

**Keywords:** Physical activity, Policy, Policy analysis, Sedentary behaviour, Framework

## Abstract

**Background:**

Policy analysis is considered essential for achieving successful reforms in health promotion and public health. The only framework for physical activity (PA) policy analysis was developed at a time when the field of PA policy research was in its early stages. PA policy research has since grown, and our understanding of what elements need to be included in a comprehensive analysis of PA policy is now more refined. This study developed a new conceptual framework for PA policy analysis – the *Comprehensive Analysis of Policy on Physical Activity* (CAPPA) framework.

**Methods:**

The development of the CAPPA framework was based on: (i) an extensive review of literature; (ii) an open discussion between the authors; (iii) three rounds of a Delphi process; and (iv) two-rounds of consultations with PA policy stakeholders.

**Results:**

The CAPPA framework specifies 38 elements of a comprehensive analysis of PA policies in the following six categories, which comprise the building blocks of the framework: (i) *purpose of analysis* (including auditing and assessment of policies); (ii) *policy level* (including: international; national; subnational; local; and institutional policies); (iii) *policy sector* (including: health; sport; recreation and leisure; education; transport; environment; urban/rural planning and design; tourism; work and employment; public finance; and research sectors); (iv) *type of policy* (including: formal written policies; unwritten formal statements; written standards and guidelines; formal procedures; and informal policies); (v) *stage of policy cycle* (including: agenda setting; formulation; endorsement/legitimisation; implementation; evaluation; maintenance; termination; and succession); and (vi) *scope of analysis* (including availability; context; processes; actors; political will; content; and effects). Based on the CAPPA framework, we also proposed broad and inclusive definitions of PA policy and PA policy analysis.

**Conclusion:**

The CAPPA framework may be used to guide future studies related to PA policy and to provide a context for the analysis of its specific components. The framework could be used in the same way for sedentary behaviour policy research. Future research should examine the extent to which PA policy analysis has covered each of the elements specified in the CAPPA framework and analyse the elements for which evidence is lacking. Future studies should also determine whether the existing tools allow for auditing and assessment of all the CAPPA elements and develop new tools if needed to allow for a more comprehensive PA policy analysis.

## Background

Insufficient physical activity (PA) is among the key risk factors for non-communicable diseases (NCDs), such as type II diabetes, some types of cancer, and cardiovascular disease [1, 2]. NCDs cause the deaths of nearly 40 million people per year world-wide, which is around 70% of overall global mortality [3]. Accordingly, insufficient PA is considered one of the main risk factors for premature mortality worldwide [4]. For example, in 2008, approximately 9% of all deaths globally were attributed to insufficient PA [5]. Insufficient PA is also associated with a range of poor mental health outcomes, such as increased risk of depression [1]. Current inadequate PA levels also generate a significant economic burden for national healthcare systems. Conservatively estimated, physical inactivity costs healthcare systems worldwide around 53.8 billion international dollars, of which 68% is paid by the public sector [6]. Given these large health and economic impacts, investing in PA promotion is widely considered a “best buy” [2, 7]. The significant public health and economic burdens of insufficient PA also emphasise the need for good public health policy related to PA.

In the last two decades, several important events have contributed to PA planning and policy development [8]. One of the initial global-level policy developments in PA promotion occurred in 2004 when the World Health Organization (WHO) issued the *Global Strategy on Diet, Physical Activity and Health* [9]. Subsequently, in 2013, the WHO published the *Global Action Plan for the Prevention and Control of NCDs* [10]. In this document, national governments, as key players in the prevention and control of NCDs, are urged to: establish national NCD targets; develop national NCD plans; and measure their progress in tackling NCDs [10]. The plan provides a menu of policy options for governments and other stakeholders to take action in NCD control and prevention and includes a global target to reduce the prevalence of insufficient PA by 10% by 2025 [10]. In 2018, the WHO launched a *Global Action Plan on Physical Activity* which recommends 20 policy actions [11] and is currently preparing a monitoring framework that will provide member states with methods to appraise progress related to PA policy development. One of the key recommended actions to support the creation of active systems is strengthening of policy frameworks, governance, and leadership systems at both subnational and national levels, to encourage implementation of actions to increase PA [11].

Increasing PA in a population requires culturally adapted, large-scale actions across whole systems, including multiple contexts, such as the health, transport, sport, urban planning, and education sectors [11, 12]. As such, one of the essential determinants of active living is the policy environment [13]. The development and implementation of policies may facilitate the creation of supportive environments for people to engage in physically active lifestyles [14, 15]. Therefore, a vital platform for developing, managing, and providing such actions is a national-level policy [12]. By developing and implementing PA policies, national governments design political and legal frameworks that are necessary for local governments and municipalities to create opportunities and environments for PA and active living [16].

It has been suggested that further research is needed to better inform future PA policy development [8, 17, 18]. Understanding the policy process and impact is essential for facilitating successful reforms [19]. A valuable tool enabling evidence-based development and improvement of policies is *policy analysis*, a “craft” that has been evolving since the 1950s [20, 21]. Policy analysis is perceived as crucial to achieving successful reforms in health promotion [19]. In relation to PA promotion, an analysis of PA-related policies can: raise awareness of current policy gaps and opportunities; demonstrate policy related actions being taken across the system; encourage important debates; contribute to meeting health objectives [22]; provide a catalyst for cooperation and communications across different sectors and levels [12]; and assist decision makers in making better informed choices in a specific problem situation [23].

In a recent systematic review, Klepac Pogrmilovic et al. [24] found more than 150 studies on national-level PA policies, which suggests that this research field is relatively well developed. However, the review also found that very few studies relied on explicit and rigorous conceptual or theoretical frameworks, which may have led to vague and/or varied definitions and conceptualisations of PA policy. The review also found that researchers in this area have not reached consensus on the definitions of PA policy and PA policy analysis [24]. Taking this into account, Klepac Pogrmilovic et al. [24] suggested that more coordinated efforts on a standardised approach to PA policy analysis would contribute to further advancement of this research area [24].

In 2002, a major consultation on PA policy development took place between the WHO and the United States Centers for Disease Control and Prevention (CDC) [25], from which a PA policy framework was recommended. The framework addressed the necessary elements that PA policy needs to encompass [25]. The first and only framework designed specifically for PA policy research was developed by Schmid and colleagues in 2006, to improve categorisation, visualisation, and understanding of PA policy research [17]. The Schmid et al.’s framework is presented as a figure with three ‘axes’: *policy*, *sector*, and *scale* [17]. The most important axis is the vertical one which presents different ways in which policy may be studied: identifying policies (i.e. description), determinants of policy, developing and implementing policy, and the impacts of policies [17]. The remaining two axes are: the *sector* axis (including: health; transportation; parks/public spaces; worksite; and school sectors) and the *levels* axis (including: local; regional; state; national; and international policies) [17]. It furthermore conceptualises public policy at three levels as: formal written codes; written standards; and unwritten social norms. The framework was developed through four stages: a literature review; a review of other policy research frameworks; collaborative discussions; and three workshops.

Schmid et al.’s framework was developed at a time when the field of PA policy research was in its early stages, and it provided a useful foundation for several studies undertaken in the field [14, 26–32]. However, PA policy research has since grown as a research area [24, 33], and our understanding of what elements need to be included in a comprehensive analysis of PA policy is now more refined. For example, the scope of Schmid et al.’s framework [17] does not cover formal processes and unwritten formal statements. Also, it is focused primarily on public policies, with less emphasis on non-governmental policies (e.g. private sector policies) related to PA. Furthermore, the framework does not: aim to provide a platform to facilitate a specific policy analysis; take into account all stages of the policy cycle at which policies may need to be studied; or acknowledge that PA policy analysis may be focused on various aspects, such as the content of a policy, the context surrounding a policy, or the actors involved in the development of a policy. Therefore, a more comprehensive framework is needed to reflect this evolving and diversifying field and to better guide contemporary and future PA policy research.

Applying a comprehensive approach to PA policy, with a focus on analysis, may strengthen the evidence base on PA policy development and content, improve comparability between studies, and provide insight into why some countries, institutions, and agencies are more successful in developing enabling contexts within which PA promotion is more likely to happen and achieve real impact. The aim of this paper was to develop a new conceptual framework for PA policy analysis – the *Comprehensive Analysis of Policy on Physical Activity* (CAPPA) framework.

## Methods

**Fig. 1:**
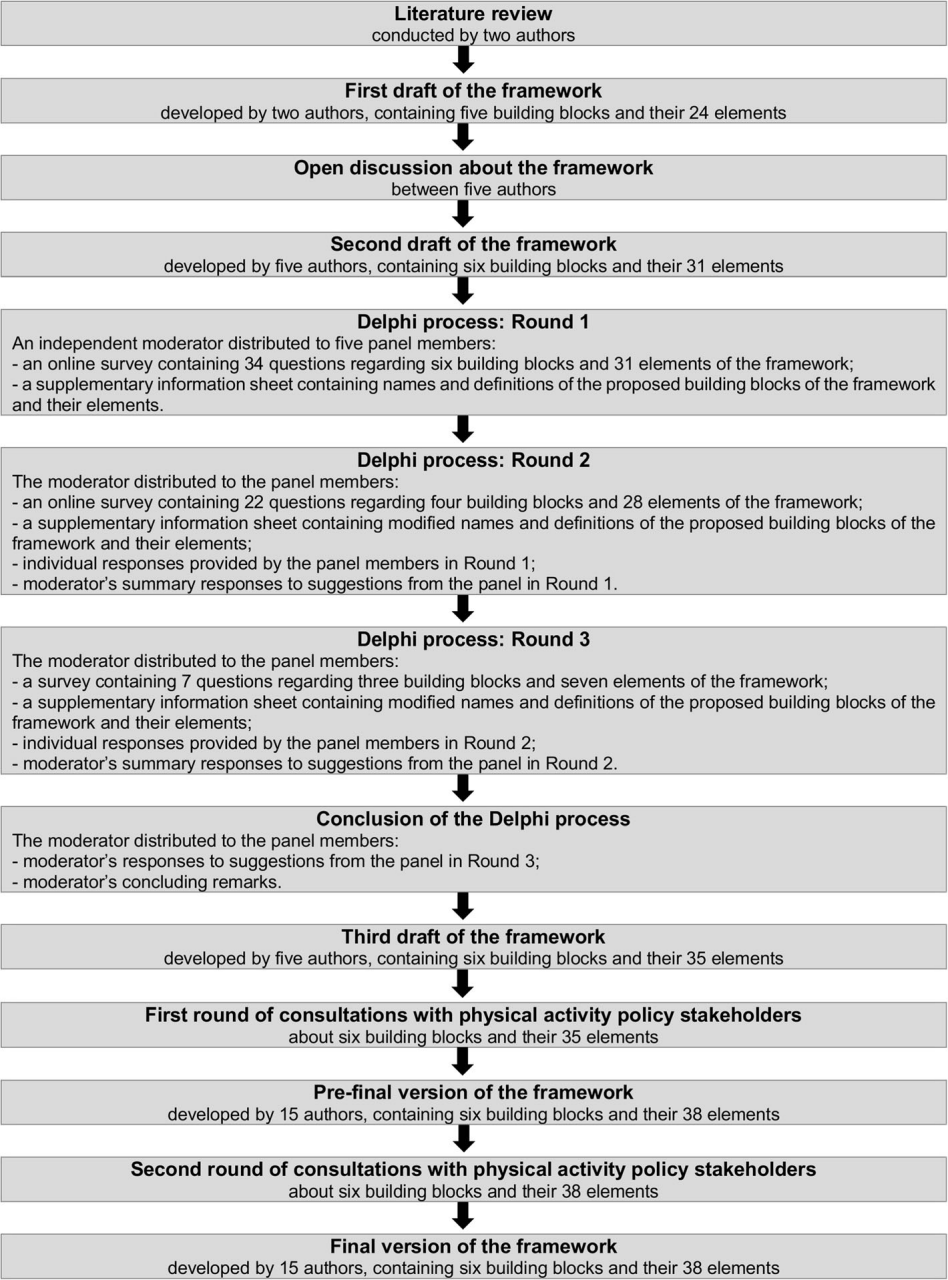
Summary of the *Comprehensive Analysis of Policy on Physical Activity (CAPPA)* framework development

The development of the CAPPA framework was based on: (i) an extensive review of literature; (ii) an open discussion between the authors; (iii) three rounds of a Delphi process; and (iv) two rounds of consultations with ten PA policy stakeholders. The development of the framework is depicted in Fig. 1. We conducted a systematic literature review to identify studies that analysed national PA and/or SB policies [24]. By reviewing the content of 203 publications included in the review, we found 25 studies that relied on a theoretical or conceptual framework. For the current study, we reviewed the frameworks cited in these studies. Additionally, we conducted an extensive search of the literature on theoretical and conceptual frameworks used for the analysis of other PA policies (not national) and other public health policies. The search was conducted through reference lists of all identified articles in the systematic literature review, authors’ own archives, and the Google Scholar database. The initial draft of the CAPPA framework was developed by two authors (BKP and ZP) through a discussion based on the theoretical models and concepts presented in the existing literature related to policy analysis in general [20, 21, 34–40] and policy analysis within the health and PA research field [12, 17, 31, 32, 41–46].

The first draft of the framework was revised on the basis of written comments provided by three authors (GOS, KM, and SJHB) and an open discussion between five of the authors (BKP, GOS, KM, SJHB, and ZP). These five authors were selected purposefully, as each one of them had specific academic expertise important for the development of the framework, including political science (BKP), psychology and qualitative methods (GOS), PA policy analysis (KM), epidemiology of PA and SB (SJHB), and methods and measurement in public health (ZP). The second draft of the framework was further considered by these authors, through a three-staged Delphi decisional process. The purpose of the Delphi process was to: (i) get independent suggestions from the panel members about how to improve the second draft of the framework; and (ii) achieve consensus about the draft framework. The Delphi method was employed to ensure anonymity in the process of reaching consensus on the structure and wording of the draft framework.

The Delphi method is a systematic approach to reaching consensus through interactive communication among experts [47]. The Delphi methodology is often used in PA policy research [48–50], as well as within the field of PA research in general [51]. Various tools may be employed to administer a Delphi process [52, 53]. For the current study, the panel members provided information by completing online surveys. An independent researcher outside the author team and the Delphi panel acted as the moderator for the Delphi process. Before starting each round, the moderator distributed to the panel members an email invitation containing the survey web link and a supplementary file with a detailed explanation of the draft structure of the framework and the definitions of the building blocks of the framework and their elements. After each round, the moderator collected the responses and provided feedback to the panel members. The feedback included summary comments related to each section of the survey as well as anonymised individual responses provided by all panel members.

The first round of the Delphi process covered three key areas: (i) name of the framework; (ii) overall structure of the framework; and (iii) the names and the definitions of all proposed building blocks of the framework and their respective elements. A combination of closed and open-ended questions was used in the survey for each specific section of the framework. For example, in the section of the survey related to the category “purpose of analysis”, these questions were posed: (i) “Do you agree with the inclusion, proposed names, and proposed definitions of the following elements of the framework in the ‘Purpose of analysis’ category?” (closed “yes/no” response); (ii) “If you disagree with the inclusion, name, and/or definition of any of the proposed elements, what would you suggest to change and why?” (open-ended response); (iii) “Do you think any other elements should be added to this category of the framework?” (closed “yes/no” response); and (iv) “If you do, please propose the names and definitions of the additional elements and briefly explain why you think they should be added” (open-ended response).

The third draft of the framework, developed in the Delphi process, was then revised through two-rounds of consultations with ten PA stakeholders, authors of the paper (AB, ARV, CW, HR, MK, MP, NC, NM, SK, and WB), who were asked to provide their written comments on the building blocks and elements of the framework and their definitions. The members of the consultation panel were selected purposefully, where the criteria for their inclusion in the panel were: (i) they have participated in the development of PA policy; and/or (ii) they are experts in PA policy research. Expertise of the consultation panel members relevant to the development of the framework included: development, implementation, and evaluation of PA policies and programmes; PA surveillance and monitoring; development of PA guidelines; PA interventions; public policy; and building international and global public health capacity. The consultation panel members were selected from various contexts, such as public policy, academia, national and international organisations for PA promotion, and public health consultancy. The comments on the third and pre-final draft of the framework made by the members of the consultation panel were discussed among all fifteen authors, which led to the development of the final version of the framework.

In this paper we relied on the broad and common definition of the term “policy analysis” that is “Policy analysis is any form of policy-relevant research” [54]. Based on the literature review and the CAPPA framework, herein we proposed definitions of PA policy and PA policy analysis that are aligned with a comprehensive approach to analysing PA policies.

## Results

The first draft of the framework, developed through the literature review and collaborative discussions of two authors, contained five categories (i.e. building blocks of the framework): *purpose*; *level*; *sectors*; *type of policy*; and *aspect of policy* and their 24 elements. The framework was modified after an open discussion and extensive comments from the remaining authors. The second draft of the framework contained six building blocks of the framework (*purpose of analysis*; *policy level*; *policy sector*; *type of policy*; *stage of policy cycle*; and *scope of analysis*) and their 31 elements. The second draft of the framework was then refined through the Delphi process. During the three rounds, panel members reached consensus on more than 40 discussion points, while the final decision on two discussion points was made by a four-fifths supermajority vote. The Delphi panel agreed on the inclusion and definitions of six building blocks of the framework and their 35 individual elements. The final version of the framework was developed through two rounds of consultations with ten PA policy stakeholders. The consultation panel members made a total of 43 suggestions. Based on the suggestions and following a discussion between all authors of the paper, 32 final changes were made to the framework. This included: (i) changing the names of four elements of the framework; (ii) modification of fifteen definitions; (iii) adding two additional elements to the framework; (iv) dividing one element into two elements; and (v) refining the examples provided for ten elements.

**Fig. 2:**
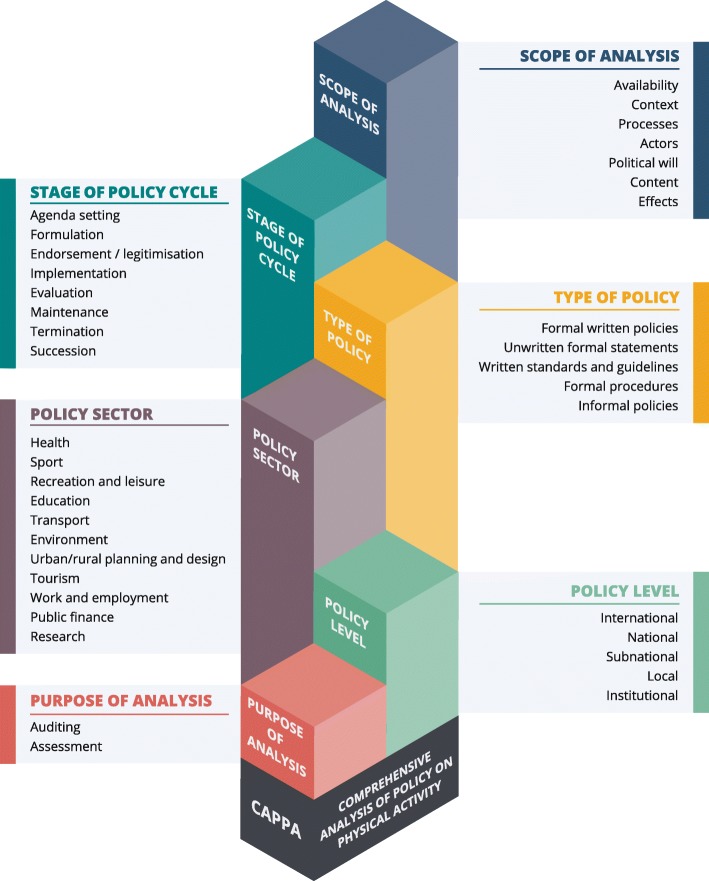
Structure of the *Comprehensive Analysis of Policy on Physical Activity (CAPPA)* framework

The final CAPPA framework (Fig. 2) specifies 38 elements of a comprehensive analysis of PA policies in the following six categories (i.e. building blocks of the framework): *purpose of analysis* (including: auditing and assessment of policies); *policy level* (including: international; national; subnational; local; and institutional policies); *policy sector* (including: health; sport; recreation and leisure; education; transport; environment; urban/rural planning and design; tourism; work and employment; public finance; and research sectors); *type of policy* (including: formal written policies; unwritten formal statements; written standards and guidelines; formal procedures; and informal policies); *stage of policy cycle* (including: agenda setting; formulation; endorsement/legitimisation; implementation; evaluation; maintenance; termination; and succession); and *scope of analysis* (including: availability; context; processes; actors; political will; content; and effects)*.* In Table 1, we provide the definitions of the building blocks and elements of the framework, together with examples that may facilitate their understanding.

**Table 1:** Definitions of the building blocks and elements of *Comprehensive Analysis of Policy on Physical Activity (CAPPA)* framework

Term	Definition, explanation and/or example
PURPOSE OF ANALYSIS	The purpose of a policy analysis
Auditing	Inquiry about a certain aspect of policy but not rating, grading, judging, or evaluating it. An example of a questionnaire item used for this purpose is: “Does Australia have a national PA strategy?”.
Assessment	Grading, rating, judging, or evaluating policy. An example of a questionnaire item used for this purpose is: “On the scale from 1 to 10, please rate to what extent is the Australian PA strategy evidence-based?”.
POLICY LEVEL	The level on which a policy was enacted and/or implemented
International	Policy that was enacted, endorsed, and/or implemented by an international political body (e.g. a policy of the United Nations).
National	Policy that was enacted, endorsed, and/or implemented by the national government or a governmental body (e.g. a policy of the Australian national government).
Subnational	Policy that was enacted, endorsed, and/or implemented below the national level but above the local level (e.g. a policy of the state government of Victoria, Australia).
Local	Policy that was enacted, endorsed, and/or implemented by a local government (e.g. a policy of the Melbourne City Council).
Institutional	Policy that was enacted, endorsed, and/or implemented by a public or private institution for its own purposes (e.g. a policy of the Melbourne High School).
POLICY SECTOR	The sector in which and/or for which a policy was developed and/or implemented
Health	The health sector includes all policies relevant to products and services for preventive, curative, rehabilitative, or palliative healthcare (e.g. a document by the U.S. Department of Health and Human Services *Physical Activity and Health: A Report of the Surgeon General,* which mentions that healthcare professionals in schools should be especially trained to gain motivational interviewing skills related to PA [55]).
Sport	The sport sector includes all policies that refer to products and services for active or passive engagement of people in sport (e.g. *Scotland’s sport strategy for children and young people – Giving children and young people a sporting chance*, which sets out Scottish Government’s vision for children and young people’s participation in sport [56]).
Recreation and leisure	The recreation and leisure sector includes all policies that refer to products and services for active or passive engagement of people in recreational exercise and other leisure-time physical activities (e.g. *Leisure Strategy and Action Plan 2015–2020* by the City of Darebin, which is a local-level document issued to direct the promotion of mental and physical wellbeing through active lifestyle [57]).
Education	The education sector includes all policies related to providing education to people in educational settings, such as childcare centres, schools, and universities (e.g. *Physical and Sport Education policy* by the State of Victoria, Australia, which states that it is mandatory for all government schools to conduct sport and physical education [58]).
Transport	The transport sector includes all policies related to the transportation of humans, animals, and goods (e.g. *Smarter Travel, A Sustainable Transport Future - A New Transport Policy for Ireland 2009–2020*, issued by the Department of Transport, Tourism and Sport, which aims to support and promote active transportation, in particular walking and cycling [59]).
Environment	The environment sector includes all policies relevant to products and services related to the built and natural environment (e.g. Swiss national *Environment and Health Action Plan,* which aims to double the number of journeys made by bicycles, as they are an example of ecologically sound and health-promoting form of mobility [60]).
Urban/rural planning and design	The urban/rural planning and design sector includes all policies relevant to the design and development of land use, the built environment, and infrastructure in and around urban and rural areas (e.g. Norway’s *the Planning and Building Act,* which mentions that configuration of physical surroundings affects the opportunities to engage in PA [61]).
Tourism	The tourism sector includes all policies relevant to attracting, accommodating, and entertaining tourists and organising travel for business and pleasure (e.g. *Switzerland Mobility* programme, a national-level set of resources for bicycling, walking, hiking, and additional activities, which also provides tourism offers [14]).
Work and employment	The work and employment sector includes all policies relevant to the workplace, paid work, volunteer work outside the volunteer’s household, employment, and retirement (e.g. A guidance document entitled *Best practices for the assessment and control of physical hazards* by the Government of Alberta, Canada, which states that workers should be encouraged to move around and stand up as much as possible [62]).
Public finance	The public finance sector includes all policies related to allocation of monetary resources (e.g. *The Victorian Budget 2018/19* which includes allocation of AUD 22.7 million to improve the active transportation network [63]).
Research	The research sector includes all policies relevant to systematic creation of new knowledge and the use of the current body of knowledge to creatively generate new outcomes. PA-related policies in this sector may indirectly affect PA in the population (e.g. Canada’s *Physical Activity and Sport Act,* which states that the Minister will take appropriate measures to assist in studies or research related to sport and PA [64] or the decision made by a Ministry of Science to allocate additional funds for research on the effectiveness of population-level PA interventions).
TYPE OF POLICY	Type of a policy according to its format (i.e. written or unwritten) and character (i.e. formal vs. informal and binding vs. non-binding)
Formal written policies	Formal written codes, strategies, plans, decisions, regulations, and directives that have been officially enacted and/or endorsed by the governing body at a given level, such as the national government at the national level or a school board at the institutional level (e.g. *Active Victoria, A strategic framework for sport and recreation in Victoria 2017–2021*, issued by the Victorian Government, Department of Health and Human Services [65]).
Unwritten formal statements	Official statements made in public by or on behalf of an official representative that were not put in writing (e.g. statement made by Senator Bridget McKenzie, the Australian Minister for Rural Health, Sport and Regional Communications, in her speech at the Australian Local Government Association’s Annual General Assembly about the commitment of the Australian Government to improve PA of people living in regional areas).
Written standards and guidelines	Written policies that guide choices, that is, they only recommend certain behaviours, practices, or processes but do not create an obligation for stakeholder adherence (e.g. *Australia’s Physical Activity and Sedentary Behaviour Guidelines*, issued by the Australian Government, Department of Health [66]).
Formal procedures	Formal actions and processes conducted or authorised by an official body or their representatives that are indicative of the body’s position or commitment regarding PA (e.g. surveillance of PA through the *Australian Health Survey* commissioned by the Australian Government is an indicator of potential commitment of the Government to support the promotion of PA [67]).
Informal policies	Informal norms, actions, voluntary codes of practice, and processes supported by an official body or their representatives that are indicative of the body’s position or commitment regarding PA (e.g. traffic police implement an informal policy based on an unwritten norm not to fine cyclists who ride bicycles on footpaths in areas where there are no designated bike paths, despite the fact that a formal written policy forbids cycling on footpaths).
STAGE OF POLICY CYCLE	A stage in the life cycle of a policy
Agenda setting	A stage in the policy cycle encompassing the processes of problem identification that require attention from the governing body at a given level (e.g. by the national government at the national level or by a school board at the institutional level). Typical examples of questions include: “What informed the agenda setting for the national PA strategy?”; “What processes were undertaken to set the agenda?”; and “Who participated in the agenda setting?”.
Formulation	A stage in the policy cycle encompassing the processes included in the development of a policy. It may involve various processes such as setting objectives, conducting consultations with stakeholders, selecting possible solutions to a problem defined in the previous stage, or estimating costs. Typical examples of questions include: “What informed the formulation of the national PA strategy?”; and “Who participated in the development of the policy?”.
Endorsement/legitimisation	A stage in the policy cycle encompassing actions and processes directed at endorsing and/or enacting a policy and ensuring that policy has a required political support. Typical examples of questions include: “Which bodies advocated for the adoption of the national PA strategy?”; “Which official body enacted the policy?”; and “How was the policy enacted, that is, did it involve legislative or executive approval or both?”.
Implementation	A stage in the policy cycle encompassing mechanisms and actions used to put a policy into practice. Typical examples of questions include: “Was the policy implemented as intended?”; “How was the policy implemented?”; and “Which bodies participated in the implementation of the policy?”.
Evaluation	A stage in the policy cycle encompassing mechanisms and actions used to appraise a specific policy and its impacts. This stage of the policy cycle should not be confused with a*ssessment* as a purpose of policy analysis. Typical examples of questions include: “Did a governmental body or an independent body appointed by the Government appraise the content of the national PA action plan?”; “What procedures are in place for evaluation of the national PA strategy?”; “Was the impact of national PA guidelines determined by an official body?”; and “What formal procedures are in place to determine the impact of the national PA strategy?”.
Maintenance	A stage in the policy cycle defined by continuation of a policy without any changes or with amendments. Typical examples of questions include: “What are the main reasons for the continuation of a policy?”; and “Who made the decision about the policy maintenance?”.
Termination	A stage in the policy cycle encompassing actions and processes related to the decision that policy will be discontinued. Typical examples of questions include: “Why was the national PA strategy terminated?”; “Which processes contributed to its termination?”; and “What are the expected consequences of the termination of the national PA strategy?”.
Succession	A stage in the policy cycle after the termination of a policy. In this stage, the policy in question may or may not be replaced by another policy. Typical examples of questions include: “Which policies replaced the national PA strategy after its end date?”; “Are all aspects of the discontinued PA strategy covered by the new policies?”; and “Why national PA strategy was not replaced with another policy after its end date?”.
SCOPE OF ANALYSIS	The subject matter encompassed by a policy analysis
Availability	Analysis of whether a policy exists or not (e.g. the presence of a national PA plan).
Context	Analysis of the economic, environmental, legal, political, social, and any other circumstances relevant to a policy or a stage of the policy cycle. Typical examples of questions about context would include: “Were there any specific economic circumstances around the development of the national PA strategy?”; “What budget has been allocated for the implementation of the national PA strategy?”; “What was the key stimulus for a policy action (e.g. the European Union encouraged its member states to develop national PA plans, decision maker’s personal involvement in sport and PA promotion, etc.)?”; “What are the dominant values held by the body endorsing the national PA strategy (secular, liberal, conservative, socialist, capitalist, etc.)?”; “What influence does private sector have on policy making process?”; and “Was the local PA policy developed based on the separation of powers doctrine?”.
Processes	Analysis of the procedures, mechanisms, and/or actions in a given stage of the policy cycle. Typical examples of questions include: “What processes did the national PA strategy have to go through to become implemented (e.g. after Minister’s proposal, the strategy was approved by the Parliament; only one ministry approved and issued the strategy; or several ministries issued the strategy but it was not sent to the Parliament etc.)?”; “Which mechanisms are in place to support the dissemination of PA guidelines (e.g. communication strategy)?”; “Which mechanisms were in place in the development stage of the national PA strategy (e.g. the national PA strategy was developed through inter-ministerial discussions and workshops with key stakeholders)?”; and “Did a development process of the national PA strategy allow for suggestions and improvements to be made?”.
Actors	Analysis of the stakeholders in a given stage of the policy cycle. Typical examples of questions include: “Which bodies proposed the national PA strategy?”; “Who were the actors involved in the development of the national PA action plan?”; “Are any non-governmental organisations assisting in the implementation of the national PA strategy?” and “What were the power relations between the actors involved in the development of the national PA strategy?”.
Political will	Analysis of the level of political support and/or commitment to a policy in a given stage of the policy cycle. Typical examples of questions include: “Does the Government hold regular discussions with the aim to support the implementation of national PA policy?”; “Did the Government demonstrate political will to support the implementation of the national PA strategy?”; and “Did any political actor in power publicly express support to the development of the national PA strategy?”.
Content	Analysis of the wording and substantive information included in a specific policy. Typical examples of questions include: “Does the national PA strategy reference specific target groups?”; “Does the national PA strategy have a clear statement on the timeframe for policy implementation?”; “Does the national PA strategy mention joint collaboration at different levels of government (e.g. local, regional, state)?”; “Are the national PA recommendations in your country fully in line with the WHO Global Recommendations on Physical Activity for Health?”; and “Is the policy content predominantly *downstream* (education, information) or *upstream* (legislation, standards, change of the environment)?”.
Effects	Analysis of the economic, environmental, public health, social, and other potential impacts of policy. Typical examples of questions include: “What kind of impact did the national PA strategy have on PA levels?” and “Were there any unintended consequences of the implementation of the national PA strategy?”.

### Definitions of PA policy and PA policy analysis

According to the CAPPA framework, PA policy is indicated by the totality of *formal written policies*, *unwritten formal statements*, *written standards and guidelines*, *formal procedures*, and *informal policies* (or lack thereof) that may directly or indirectly affect community- or population-level PA. Accordingly, we defined PA policy analysis as any kind of policy-relevant research that audits or assesses one or more aspects of PA policy.

## Discussion

In this study, we developed the CAPPA framework as a conceptual inventory of components necessary for a comprehensive analysis of PA policy, including definitions of two different purposes of analysis, five policy levels, eleven policy sectors, five types of policy, eight stages of policy cycle, and seven elements that reflect the scope of policy analysis. The framework was developed to improve the comprehensiveness and contribute to the standardisation of PA policy analysis research. This comprehensive conceptual framework may serve as a “road map” for researchers and academics interested in PA policy analysis as well as to policymakers and health policy practitioners interested in the development, monitoring, implementation, and analysis of PA policies. The framework can also be used for categorising PA policies or as a classification system for PA policy research. To further facilitate the standardisation of PA policy research, we also proposed definitions of PA policy and PA policy analysis that are aligned with the CAPPA framework.

### Purpose of analysis

Studies can be conducted with the purpose of *auditing* and/or *assessment* of PA policies. Policy auditing is a prerequisite for policy assessment, as we first need to know which aspects of policy exist (or existed), before we can assess them. An *assessment* of the aspects of policy identified in the audit process will then determine how good they are against certain standards. For a comprehensive analysis of PA policies, it is important to both audit and assess relevant policies. For example, a country may have a range of national PA policies in place, including a PA strategy and a PA action plan, but it is possible that none of them are evidence based, none of them specify clear targets, none of them define feasible ways to improve population-levels of PA, and none are funded or implemented. Policy *assessment* may need to be done to elucidate some of the important questions about PA policies. It should be noted, however, that policy *auditing* and policy *assessment* may be extremely time-consuming, and it is, therefore, often not practical to conduct both within a single study.

### Policy level

PA policies can be developed at various levels. The simplest classification found in the literature makes a distinction between PA policies that occur at the national and international levels [68]. PA policies at the national level are usually developed by the Government or a governmental body, but they may also be developed by non-governmental or advisory bodies, and later endorsed by the Government. The ways to classify policies below the national level may vary depending on the country in question and its political system. Policies can be developed and implemented on *subnational* levels such as state, federal, municipal, regional, and provincial. The CAPPA framework was developed with the intention of being as applicable as possible to various political systems. Therefore, we did not distinguish between a range of different levels that are below the national level and above the local level. Instead, we encompassed all such levels with the broad term “subnational”. PA policy researchers should, however, clearly distinguish between different *subnational* levels in the context of the political system they are investigating and endeavour to analyse policies separately at each of the levels. Schmid et al.’s conceptualisation of *scale* (i.e. equivalent to *policy level* in the CAPPA framework) does not include the “institutional” level, because their framework focused mainly on public policies [17], that is, the policies related to government actions [34]. In the CAPPA framework we included the “institutional” level, because policies at this level often have a key role in the development and implementation of PA interventions. Furthermore, it can be assumed that policies at one level may influence the adoption and shaping of policies at other levels. For a complete understanding of PA policy, it is therefore important to analyse policies at all levels, as well as to consider their possible interactions.

### Policy sector

Policies in a range of sectors may directly or indirectly affect PA levels in the population [41, 69]. This is also acknowledged in the Schmid et al.’s framework [17], which includes five sectors: health; transportation; parks/public spaces; worksite; and school. In the CAPPA framework we built on Schmid et al.’s sectors and added other sectors that were previously identified as relevant to this research field such as: public finance; research; sport; recreation and leisure; and tourism [11, 17, 41, 69].

It should be noted, however, that policy sectors may be termed differently and overlap more or less, depending on the specific context of a given country. Therefore, the CAPPA sectors should be interpreted in the context of a specific country. Furthermore, we acknowledge that PA policies can, and in many cases should, be cross-sectoral, that is, developed and/or implemented across multiple sectors. When classifying a policy according to the CAPPA framework, one should, therefore, not necessarily try to fit it within a single sector. This may present a methodological challenge in some classifications, but it is inevitable due to the complex nature of PA policies. Future users of the CAPPA framework may choose to report on all sectors to which a policy applies or to prioritise the sector that initiated or is responsible for the policy. For example, in the case of a *Walk to school policy* issued by the Ministry of Education, the priority could be given to the education sector, but a policy analyst could choose to report that this policy also belongs to the transport sector. When making such classifications, it is, therefore, important to clearly describe the criteria that were applied.

We also aimed to clearly differentiate between “sectors” and “settings”, because one sector usually includes multiple settings and one setting can belong to multiple sectors. For example, the education sector includes settings such as childcare centres, primary schools, secondary schools, and universities. At the same time, each of these settings is also a part of the work and employment sector, because they employ their staff. There is a vast number of settings that might include PA-related policies, and any attempt to list them all is unlikely to result in an exhaustive inventory. For this reason, in the CAPPA framework we did not provide a list of settings that are potentially relevant from the perspective of a comprehensive PA policy analysis. PA policy researchers should consider analysing PA policies in all the sectors included in the CAPPA framework and in as many relevant settings as possible.

### Type of policy

There are different types of policies, and they are not necessarily always in the written form. This has already been acknowledged by Schmid et al. [17]. They conceptualised policy at three levels: (i) formal written regulations, codes, or decisions bearing legal authority; (ii) written standards that guide choices; and (iii) unwritten social norms [17]. *Formal written policies* in the CAPPA framework correspond to Schmid et al.’s first level. *Written standards and guidelines* and *informal policies* in the CAPPA framework correspond to the second level and the third level in the Schmid et al.’s framework [17], respectively. As suggested by Schmid et al., *informal policies* are “considered to be part of culture rather than explicit policy and not a primary focus of initial physical activity policy research” [17]. However, analysing *informal policies* could bring additional valuable insights into overall PA policy directions that may subsequently inform policy decision-making. Policy may be conceptualised in a broader sense to also include *formal procedures* [44] and *unwritten formal statements* [35], which has been acknowledged in the CAPPA framework. Such statements may play an important role in shaping the general policy context within which the dominant beliefs may subsequently get converted into formal written policies. *Formal procedures,* such as PA surveillance, may be indicators of the body’s position or commitment regarding PA. Formal procedures are usually (but not necessarily) supported by a formal written or unwritten policy. Furthermore, the analysis of *unwritten formal statements* may also provide valuable insights about the intentions of a given body regarding PA. The definition of *unwritten formal statements* in the CAPPA framework is in line with the definition of public policy as an “authoritative statement by a government about its intentions” [35]. *Unwritten formal statements* related to PA have previously been studied mainly using discourse analysis as a research method [70, 71].

Investigating understudied types of policies may help better elucidate policy-related correlates of PA. For example, a conclusion that a certain country has an underdeveloped PA policy simply based on an analysis that showed it lacks *formal written policies*, may be misleading. The country might have *informal policies* in place that promote PA, and *unwritten formal statements* created through announcements or verbal declarations by its decision makers may indicate the government has well-conceived plans and mechanisms for PA promotion. In a different example, a country might have a well-developed *formal written policy*, but certain *informal policies* and *unwritten formal statements* (or lack thereof) may indicate a lack of political will to support PA promotion. It is important to note, however, that analysing *unwritten formal statements* and *informal policies* could be challenging, as they may be more difficult to identify and evaluate than *formal written statements, written standards and guidelines*, and *formal procedures*.

### Stage of policy cycle

The list and definitions of stages of policy cycle in the CAPPA framework, were mainly informed by the health policy and political science literature. The concept of policy cycles was originally “employed prescriptively as a way to organize policymaking”, but it further evolved as a framework common for analysing policies [38]. The WHO specified the following stages of the policy cycle: problem identification and agenda setting; policy formation; adoption; policy implementation; and policy evaluation [42]. Informed by Cairney’s conceptualisation of the policy cycle [38], for the purpose of the CAPPA framework we adapted the WHO’s five-stage policy cycle to include an additional three elements ─ maintenance, termination, and succession. The CAPPA framework contains eight stages which is an important advance from the four-stage structure of Schmid et al.’s earlier framework [17].

It should be noted that a policy will not necessarily go through all the stages of the policy cycle. For example, a policy may be enacted by Parliament, but that does not necessarily mean it will ever be implemented in practice. Furthermore, stages in the cycle of a given policy may not necessarily be in the order presented in the CAPPA framework. For example, some policies may be formulated without going through the *agenda setting* stage. Some policies may be formulated, maintained, and terminated without ever being implemented or ever being evaluated. Furthermore, a policy may pass multiple times through the same stage (e.g. a policy can be evaluated on several occasions). For a thorough understanding of a PA policy, it is important to analyse all the stages that it went through.

### Scope of analysis

Most previous research on national PA policies has focused on analysing *availability* of policies (i.e. whether specific policies exist) and their *content* (i.e. what information they include) [24]. Analysis of *availability* of policies should not be confused with *auditing* as a purpose of PA policy analysis, because theoretically the availability of policies can be both audited (e.g. using the open-ended questionnaire item: “Please list the PA policies that are available in your country!”) and assessed (e.g. using the question: “How would you rate the range of PA policies available in your state compared to the national level?”, with the response scale: “Less available policies” / “Similar number of available policies” / “More available policies”). The analysis of policy *content* should not be confused with *assessment* as a purpose of PA policy analysis, because the content of a policy can also be both audited (e.g. using the question “Does the national PA strategy include specific targets for different population groups?”, with the “yes/no” response scale) and assessed (e.g. with the question: “On a scale from 0 to 10, please rate the overall quality of the national PA strategy”). For some types of PA policy, the analysis of *content* can be performed by using qualitative methods for content analysis, that is, by coding and interpreting text of written documents, transcribed oral communications, and graphics.

Furthermore, Walt’s simple health policy analysis framework distinguishes between four elements: *context, content*, *process*, and *actors* [45]. Context, content, processes, and actors often play pivotal roles in different stages of the policy cycle. In the CAPPA framework, we therefore acknowledge the importance of analysing all these elements in addition to the *availability, political will,* and the *effects* of policies. Political will represents a bridge between public health action and knowledge [72] and is considered to be essential for making changes in public health policy [73]. Political support and commitment to a PA policy are recognised as highly relevant factors for the success of the policy and are, therefore, important parts of a comprehensive PA policy analysis [69, 74–76]. Researchers may be deterred from analysing the effects of PA policies, because these may be difficult to measure. It has therefore been suggested, as one of the key priorities for the progress of the PA policy research field, to develop better tools for analysing the effects of policies [17]. This was recognised by the Physical Activity Policy Research Network (PAPRN) in the USA, which conducted a ten-year study of the effectiveness of policies to increase levels of PA [77]. In 2017, they concluded there is a lack of studies on the outcomes of PA policies [78].

When it comes to an overall policy-making process, political power is often a vital force. In the political arena, various groups exercise their political power to reach their goals, either by advocating for a change or blocking it [79]. In health policy analysis, power is usually considered in relation to two elements of the CAPPA framework; namely, *processes* and *actors* [22]. However, power can also be studied within other elements of the *scope of analysis* category such as *political will* or *context*. *Political will,* necessary to introduce any policy change, may be highly influenced by power relations and values within and outside of the government. For example, members of the government can have a strong political will to increase resources necessary for the implementation of a nutrition and PA strategy that aims to reduce children’s obesity rates. However, powerful food industry lobbies may block the strategy implementation, if the proposed measures are not in their best interests.

### Definitions of PA policy and PA policy analysis

Within the field of political science, there is no consensus on what constitutes “a policy” or a policy analysis [24]. Similarly, within the PA research field, “PA policy” was defined and conceptualised differently across studies, whilst a large majority of the studies on national PA polices did not explicitly state how they defined PA policy [24]. The majority of studies that provided their operational definition of PA policy conceptualised policy within Schmid et al.’s first level [24], that is, as the formal written regulation, code, or decision bearing legal authority [17] which corresponds to *formal written policies* in the CAPPA framework. For example, several PA policy studies relied on the definition of a policy that conceptualises ‘policy’ as a ‘policy document’, that is, “a written document that contains strategies and priorities, defines goals and objectives, and is issued by a part of the administration” [31, 32, 69]. Restricting the conceptualisation of PA policy only to “written documents” may be practical for researchers, because these types of policies are usually the easiest to identify. However, this approach may exclude other possible aspects of policy such as “unwritten statements”. Some health and PA policy researchers based their studies on a broader definition of policy, which besides formal statements also includes informal institutional procedures, arrangements, and justifications for action [44]. We acknowledge that various studies have different purposes and may therefore employ the most suitable definition for the scope of the study. We also acknowledge that it may be impossible to analyse all aspects of PA policy in a single study and that sometimes it may be necessary to reduce the analysis to only one or two aspects of PA policy. However, we believe a comprehensive standardised definition of PA policy may contribute to further development of the PA policy research field. Therefore, based on the CAPPA framework and various understandings of PA policies that were detected in our recent systematic scoping review [24], we defined PA policy broadly, to be as inclusive as possible. We used a similar comprehensive and inclusive approach in defining PA policy analysis, whilst relying on the CAPPA framework and a broad definition of policy analysis from the field of political science [54].

### Possible applications of the CAPPA framework in PA policy research

The CAPPA framework can be used for a variety of purposes. These include (but are not limited to): (i) to help PA policy researchers conceptualise their study questions, that is, as a source of ideas what can and should be analysed; (ii) as a benchmark for evaluating what has been done in terms of PA policy research overall, in its specific areas, and in specific contexts (e.g. in specific countries); (iii) as a guide for policymakers, who want to influence population-level PA, on which types of policies and which policy sectors they can focus on in their endeavours; (iv) to help PA policy researchers improve between-study comparability, particularly by using the definitions provided within the framework; (v) to help assess the comprehensiveness and content validity of the available tools for PA policy analysis; and (vi) to guide the development of new PA policy analysis tools, particularly regarding the facets of PA policy they are intended to measure.

A practical example of a possible application of the CAPPA framework can be found in our recent systematic review of instruments for PA policy analysis [80]. For every instrument included in the review we determined whether it was designed for *auditing* or *assessment* of PA policies, which *policy sectors*, *types of policy*, and *stages of policy cycle* it covers, and what is encompassed in its *scope of analysis*. The list of elements of the CAPPA framework served as a benchmark for the assessment of comprehensiveness of the included instruments. An extract (for four sample instruments) from the data extraction table can be found in Table 2. The definitions provided in the CAPPA framework enabled us to conduct the assessments consistently across all instruments and between two authors who took part in the data extraction process. In the same review, we used the CAPPA framework also to guide the synthesis of findings. It enabled us to easily identify which elements needed for a comprehensive analysis of PA policy cannot be analysed using the available instruments.

**Table 2:** An example of a possible application of the *Comprehensive Analysis of Policy on Physical Activity* (CAPPA) framework: an extract from a review of instruments for the analysis of physical activity and/or sedentary behaviour policies

Instrument	CAPPA elements covered by the instrument				
	Purpose of analysis	Policy sector	Type of policy	Stage of policy cycle	Scope of analysis
*Health enhancing physical activity (HEPA) policy audit tool (PAT)*, [12, 61, 81, 82]	Auditing Assessment	Education Environment Health Sport Recreation and leisure Tourism Transport Urban planning and design Work and employment	Formal written policies Written standards Formal procedures	Formulation Implementation Evaluation Maintenance	Availability Context Processes Actors Political will Content
*A Graphical, Computer-Based Decision-Support Tool to Help Decision Makers Evaluate Policy Options Relating to Physical Activity* [74]	Assessment	None	None	Formulation Implementation	Context Effects Political will
*Global Observatory for Physical Activity (GoPA!) questionnaire* [83, 84]	Auditing	None	Formal written policies Formal procedures	None	Availability
*Analysis of Determinants of Policy Impact* [44, 85]	Auditing Assessment	None	Formal written policies Formal procedures	Formulation Implementation Evaluation	Context Processes Actors Content Effects

### Applicability of the CAPPA framework to the analysis of sedentary behaviour policy

Research suggests that uninterrupted prolonged periods of sedentary behaviour (SB) (i.e. waking activities in a sitting, reclining, or lying posture with very low energy expenditure) are associated with increased risk of cardiovascular disease, type II diabetes, and some types of cancer [86]. It was estimated that high SB is responsible for nearly 4% of deaths from all causes internationally [5]. It is therefore of public health importance to reduce SB in the population. PA and SB are often considered within the same study, as these behaviours are co-dependent [87]. A recent review found that all but one study that analysed national SB policies also analysed PA policies [24]. Given that PA and SB policy research fields largely overlap and that contexts of PA and SB policies are very similar, the CAPPA framework and definitions analogous to the ones provided for PA policy and PA policy analysis may also be used to guide research on SB policies.

### Strengths and limitations of the study

The key strength of this study is a rigorous method used to develop the framework, which included an extensive literature review, three rounds of Delphi process, and two rounds of consultations with stakeholders. The CAPPA framework provides a categorisation of a complex area into measurable component parts. Each of these components is defined, and can be audited and assessed in combination to provide a comprehensive understanding of PA policy. The main strengths of the CAPPA framework are its: (i) comprehensiveness; (ii) generalisability to different political contexts; (iii) supporting definitions that underpin each building block of the framework and its elements; and (iv) visual simplicity.

The CAPPA framework is also subject to some limitations. The authors aimed to make the building blocks of the framework and their elements as generalisable as possible, but given a variety of policy contexts internationally, some elements may not be applicable to all countries. Also, due to the complexities in the political context, an overlap between the various elements of the framework was inevitable. Future users of the framework should acknowledge the possible overlap and specify the way they choose to deal with it. Whilst the first draft of the framework was developed based on a comprehensive literature review, due to the wealth of literature in the fields of political science, health policy research, and PA policy research, the authors acknowledge there might be aspects of PA policy analysis that are not encompassed by the CAPPA framework.

## Conclusion

The CAPPA framework may be used to guide future studies related to PA policy, provide a context for the description, understanding, and analysis of its specific components and serve as a classification system for research on PA policies. It may also serve as a benchmark for the evaluation of comprehensiveness of existing tools for the analysis of PA policy and guide the development of new tools. The framework can be used in the same way for SB policy research. Operational definitions of different aspects of policy varied significantly across previous studies in this area [24]. The definitions of specific types of policy, aspects of policy, and purposes of policy analysis provided in the CAPPA framework might help in achieving standardisation of terminology in this area and in improving the comparability of findings across different studies. Future research should examine the extent to which PA policy analysis has covered each of the elements specified in the CAPPA framework. Future studies should also evaluate whether the existing tools for PA policy analysis allow for auditing and assessment of all the elements of the CAPPA framework and develop new tools where needed.

## Data Availability

Not applicable.

## Abbreviations

CAPPA: Comprehensive Analysis of Policy on Physical Activity
CDC: Centers for Disease Control and Prevention
NCD: Noncommunicable disease
PA: Physical activity
SB: Sedentary behaviour
WHO: World Health Organization
